# Between Difference and Belonging: Configuring Self and Others in Inpatient Treatment for Eating Disorders

**DOI:** 10.1371/journal.pone.0105452

**Published:** 2014-09-11

**Authors:** Karin Eli

**Affiliations:** Unit for Biocultural Variation and Obesity, Institute of Social and Cultural Anthropology, University of Oxford, Oxford, United Kingdom; UNSW Australia, Australia

## Abstract

Dedicated inpatient care for eating disorders has profound impact on patients' embodied practices and lived realities. Analyses of inpatients' accounts have shown that participants endorse complex and conflicting attitudes toward their experiences in eating disorders wards, yet the apparent ambivalence that characterizes inpatient experiences has not been subject to critical examination. This paper examines the narrated experiences of 13 participants (12 women and one man; age 18–38 years at first interview) with past or present anorexia nervosa, bulimia nervosa, or eating disorder not otherwise specified, who had been hospitalized in an inpatient eating disorders ward for adults in central Israel. The interviews, which took place in 2005–2006, and again in 2011, were part of a larger longitudinal study exploring the subjective experiences of eating disorders and recovery among Israeli adults. Employing qualitative analysis, this study finds that the participants' accounts were concerned with dynamics of difference and belonging, as they played out in various aspects of inpatient care, including diagnosis, treatment, relationships with fellow patients and staff, and everyday life in hospital. Notably, participants simultaneously defined themselves as connected to, but also distinct from, the eating disordered others who formed their reference group at the ward. Through negotiating a protectively ambivalent positioning, participants recognized their eating disordered identities and connected with others on the ward, while also asserting their non-disordered individuality and distancing themselves from the potential dangers posed by ‘excessive’ belonging. The paper suggests that this ambivalent positioning can usefully be understood through the anthropological concept of *liminality*: being both a *part of* and *apart from* one's community.

## Introduction

Dedicated inpatient care for eating disorders has profound impact on patients' embodied practices and lived realities. Marked by the strict regulation of patients' dietary intake and physical activity, the routine measurement of patients' physiological markers (e.g. body weight, blood and urine samples), and the continuous surveillance of patients' engagement in seemingly mundane activities (e.g. nursing staff observing patients as they watch television programmes in the common room), inpatient treatment for eating disorders alters patients' everyday ways of being-in-the-world.

A number of qualitative studies have analyzed the treatment experiences of women and adolescent girls who had been hospitalized in inpatient eating disorders wards. Some studies focused on specific elements of treatment, such as mealtime (Long et al., 2012 [1]) and bed rest (Griffiths et al., 1998 [2]), while others emphasized the experience of treatment as a whole (Colton and Pistrang, 2004; Offord et al., 2006; Tierney, 2008; Smith, 2012 [3] – [6]). The latter set of studies identified similar themes that characterized participants' experiences, such as finding support and connections on the ward (cf. Rich, 2006 [7]), and preferring individualized care that involved patients in decision making. However, participants did not necessarily endorse similar attitudes; indeed, they experienced the same institutional practices and structures in sometimes starkly opposed ways. Colton and Pistrang (2004 [3]) describe conflict and ambivalence as inherent to the experiences described by their study's participants. They state that, while participants spoke of feeling supported by the patient community on the ward, they also said they felt triggered by their fellow patients; and while participants said they felt the ward's regime was helpful, they also said the regime ‘punished’ them (Colton and Pistrang, 2004 [3]). Likewise, while Smith (2012 [6]) found that participants felt inpatient treatment provided them with a safe environment, free of the stressors they encountered in the ‘outside’ world, she also found that they were concerned that the inpatient environment constituted an abnormal space, which did not prepare them adequately for recovery.

The complex and conflicting attitudes expressed in participants' accounts of inpatient experiences have been explained as reflecting participants' differing places on the ‘stages of change' scale (Colton and Pistrang, 2004 [3]), or as the result of variations in individualized care and community support (Smith, 2012 [6]). Other studies have suggested that ambivalence is a central feature of eating disorders, and that the ambivalent attitudes that people with eating disorders hold are rooted in the conflation of illness and identity, and in emotional and perceptual obstacles to motivation for recovery (Reid et al., 2008; Williams and Reid, 2010; Nordbø et al., 2012 [8] – [10]). These explanations, while highlighting the important role that each person's subjective and relational circumstances play in the formation of patient experience, do not examine ambivalence as situated in the unique social realities and practices patients encounter at the ward.

Ethnographic studies of eating disorder inpatient wards suggest that institutional structures and practices, far from being therapeutic givens, are deeply socially embedded. Indeed, some scholars argue that the same social logics that act in eating disorders are also displayed in the institutions meant to treat them. In her analysis of an inpatient ward in the United States, Gremillion (2002 [11]) argues that ward practices enact broader societal idealizations of ‘proper’ femininity, individuality, the family, and fitness, such that treatment itself – with its focus on measuring, exactness, surveillance, and resistance – reproduces the very practices that constitute anorexia nervosa. Similarly, analyzing the construction of space in an Australian inpatient ward, Warin (2005 [12]) suggests that, with its inversions of public and private (e.g. washing is subject to surveillance, but eating is conceptualized as an act to be undertaken in the solitude of the hospital ‘bedroom’), and its enmeshment of such disparate acts as eating, sleeping, and purging/excreting (all potentially performed in the same room), the ward re-enacts the patients' own spatial configurations of anorexic life. Other analyses have highlighted how inpatient treatment for eating disorders implicates locally-salient modes of selfhood. In Gooldin's (2002 [13]) ethnography of an inpatient ward for adolescents in Israel, she argues that broader social constructions of normative, productive women as soldiers and mothers are actively woven into the clinicians' motivational discourses and practices on the ward, highlighting recovery as a means to assume these desired roles. Comparatively analyzing practices in inpatient wards in Mexico and the US, Lester (2007 [14]) argues that therapeutic paradigms are locally-specific; she draws attention to the Mexican ward's focus on ‘codependency’ and the US ward's focus on ‘individuation’ to demonstrate how each situates eating disorders within local understandings of personhood, gender, and agency.

Drawing on insights from both the patient experiences and the ethnographic literatures, this paper aims to identify the ways in which inpatient ambivalence might be embedded in the special social institutional setting that an eating disorders ward presents, beyond patient-specific motivation for recovery. Such analysis is of particular importance given the high ‘drop out’ rates of people with eating disorders from inpatient programmes (20.2% – 49.6%) (Wallier et al., 2009 [15]), and the limited success of research that has, so far, exclusively focused on patient-specific risk factors for ‘dropping out’, such as personality characteristics and body mass index (Wallier et al., 2009; Kahn and Pike, 2001; Surgenor, McGuire, & Beaumont, 2003 [15]– [17]). This paper is based on research carried out as part of a longitudinal medical anthropology study concerning the subjective experience of eating disorders in Israel (2005–2006; 2011). The first phase of the study constituted the author's doctoral research, and the second phase was part of the author's postdoctoral research; in both phases, the author was centrally concerned with exploring how identity is embedded in the sensory experiences of eating disorders and recovery. The current paper focuses on a subsample of participants who were hospitalized in a dedicated eating disorders ward for adults in central Israel. While these participants' accounts of inpatient care were part of their greater narratives of eating disorders, their experiences as inpatients present a substantively unique set of themes that merits separate consideration.

## Methods

### Participants

This paper is based on data collected from interviews with 13 participants, who took part in a larger, longitudinal medical anthropology study concerning the subjective experience of eating disorders in Israel (2005–2006, n = 36; 2011, n = 23). The 13 participants comprised a subset of the total purposive sample, which targeted people who had eating disorders, regardless of treatment experiences. All 13 participants were hospitalized in the same inpatient eating disorders ward for adults in central Israel. One additional participant was also hospitalized in the same ward; however, since this participant said she was traumatized by her multiple hospitalization, the author chose not to pursue further discussion of her inpatient experiences, and therefore excluded her from this subsample. Of the 13 participants included in the present paper, nine were interviewed again in 2011; two of the participants interviewed in 2011 were hospitalized between the two phases of the study. There was no notable difference in hospitalization-related themes between the four participants who were interviewed only in 2005–2006, and those who were interviewed again 2011.

The 13 participants included 12 women and one man, who ranged in age from 18 to 38 at the time of first interview (2005–2006). Twelve participants were hospitalized with a diagnosis of anorexia nervosa or its subclinical variants (eating disorder not otherwise specified, as defined by the *DSM-IV-TR*) (APA, 2000 [18]), and one was hospitalized with a diagnosis of bulimia nervosa. The author recruited the participants through a number of sources: an outpatient eating disorders clinic (four participants), an online pro-recovery eating disorders forum (two participants), an eating disorders advocacy organization (one participant), and an informal network of participants recruited through chain-referral/snowball sampling by a key participant (six participants).

The recruitment process included several steps. The informal network participants were first contacted by a key participant, with whom they were acquainted through shared eating disorders environments (mostly clinical or online). The author contacted these participants after they had given their initial agreement to the key participant, explaining the study to them and what it would entail, and asking them afterwards whether they would agree to participate. The participants who were recruited through the discussion board were not approached directly. The author posted three public messages concerning the study, inviting anyone who would like additional information to send a confidential message to her through the discussion board's private messaging system. Discussion board participants were recruited following message exchanges and telephone conversations, during which the author used the same explanatory recruitment process as she did with the other participants. With respect to the participants recruited through the clinic, staff initially identified and contacted former and current patients whom they judged fit to participate in the study. After the potential participants had agreed to share their contact details with the author, she called them to explain the study further and ask if they might want to participate. In all cases, the author explained that the study would involve interviews about the participants' personal experiences of eating disorders, with a view to cultural issues that might be particular to Israel.

### Ethics statement

The first phase of the study was approved by the University of Oxford's Social Sciences and Humanities Inter-Divisional Research Ethics Committee (under the auspices of the University of Oxford's Central University Research Ethics Committee; June 2005), and was also approved by the Israeli Kupat Holim (health-care fund) Helsinki Ethics Committee (August 2005). The second phase of the study received approval from the University of Oxford's Social Sciences and Humanities Inter-Divisional Research Ethics Committee (February 2011), and did not require separate Helsinki Ethics Committee approval. In order to ensure informed consent, the author explained the study to all potential participants before asking them whether they wished to participate, and provided detailed information sheets and consent forms at the beginning of each interview, either in Hebrew or in English. The participants provided written consent for this study. All names used in this paper are pseudonyms, and identifying details have been concealed or altered to ensure confidentiality.

### Data and analysis

Data for the study were collected through in-person semi-structured interviews. The majority of initial interviews during the first round of fieldwork opened with the same question (‘when and how did it begin?'), which framed the participants' narratives. The author then followed with questions that further explored each participant's initial narrative, with emphasis on sensory experience (e.g. ‘how did you feel when you ate?’) and meaning-making (e.g. ‘why do you think you expressed yourself through food?’). During the second round of fieldwork, the author opened each interview by asking each participant what she remembered about the time in her life when the first interview took place; the interviews then focused on exploring the unfolding of recovery and chronicity processes over the intervening years, and the meanings thereof. Interviews ranged in length according to each participant's preferences and availability, ranging from approximately 30 minutes to 4 hours in length. However, some participants were interviewed more than once within the same interview cycle, such that, for example, the participant whose first interview lasted 30 minutes was then interviewed two weeks later for an additional hour.

The study was designed to include people outside inpatient settings, and most interviews were thus retrospective, taking place months or years following hospitalization. Accordingly, the majority of interviews took place either in participants' homes or in public places of their choice (cafes and parks). However, two participants had been hospitalized in the eating disorders ward during the period of the study, and the author met with them on site. As both had multiple experiences of hospitalization at the ward, their narratives included a mix of retrospection and immediacy.

The interviews were digitally recorded and supplemented with written notes; the author transcribed the recordings, and translated the transcripts from Hebrew to English; during the process of translation, the author noted Hebrew-specific phrasings (e.g. ‘menu’ [dietary regimen], ‘being without a period’ [amenorrhea], ‘going up’ [gaining weight]) to make sure that interpretations accurately reflected locally nuanced meanings.

For this paper, the author used a modified form of interpretative phenomenological analysis (IPA) to explore the participants' subjective experiences of inpatient care (Smith et al., 1999; Smith and Osborn, 2003 [19] [20]). The analysis is grounded in a phenomenological and contextual framework, but as the typical idiographic level in anthropology consists of small-scale groups, the author coded and clustered the themes across the corpus of interviews, rather than case by case, while still being attentive to variations between, and patterns within, the experiences of individual participants. Working with the original Hebrew transcripts, the author first extracted and collated the sections of the transcripts that concerned adult inpatient treatment. The author began analyzing these collated extracts by reading and re-reading them several times, with the initial purpose of identifying and denoting participants' accounts of positive and negative experiences of hospitalization. The larger focus on ambivalence emerged from these initial readings and annotations, which revealed the co-existence of positive and negative experiences in the majority of the participants' narratives. The author returned to the transcripts, coded for the subthemes that emerged in each interview, and then re-coded the transcripts de novo, looking for these emergent subthemes again in the entire corpus (examples of subthemes included: ‘helping other patients'; ‘feeling observed by other patients when eating’; ‘feeling able to defer coping’). The majority of subthemes related to participants' interpretations of their experiences on the ward, and were thus included in the analysis; a few subthemes related to non-interpretative descriptions of life on the ward (e.g. the daily schedule, the lunch menu), and were thus excluded.

The subthemes were then clustered and classified under super-ordinate themes; these were further classified into three thematic categories. The thematic categories and super-ordinate themes are included in the results section. The author tabulated the subthemes and super-ordinate themes on a spreadsheet, and returned to the transcripts to record their occurrence in the interviews of each of the participants. This allowed for the validation of the recurrence of themes, and assured that data were accurately represented in the analysis, in accordance with each theme's salience for the sample.

A chronological ordering of the tabulated themes (by dates of interview sets) revealed that only two new subthemes were introduced in the last two sets of interviews. Notably, one of these subthemes concerned hospitalization after giving birth – a unique experience in this sample. As the majority (∼80%) of subthemes occurred in interviews with the first seven participants, and given the relative homogeneity of the group (all participants having been hospitalized in the same ward), it is possible to conclude that saturation was reached (cf. Guest et al., 2006 [21]).

The findings are interpreted with particular attention to explicating ambivalence in and conflict between patients' experiences, as contextualized within the unique social milieu of the ward. The focus on ambivalence emerged during the analytic process. The author's interpretive stance, which contextualized ambivalence in the ward's social milieu, was influenced by her training and positioning as an anthropologist.

## Results

The following three thematic categories emerged in the analysis, each divided into two super-ordinate themes: a sense of recognition and legitimacy (‘the eating disordered self’ and ‘the patient as an individual’), dynamics within the inpatient community (‘seeing the self in others’ and ‘dangerous others’), and boundaries vis-a-vis the ‘outside’ world and one’s own illness (‘sheltering boundaries’ and ‘oppressive boundaries’) (see Table 1 for summary). Running through these thematic categories was a central concern with the negotiation of identity during and through hospitalization.

**Table 1:** Inpatient experiences: A summary of thematic categories and super-ordinate themes (n = 13).

Thematic categories	Super-ordinate themes
A sense of recognition and legitimacy (n = 9)	The eating disordered self (n = 4)
	The patient as an individual (n = 8)
Dynamics within the inpatient community (n = 12)	Seeing the self in others (n = 9)
	Dangerous others (n = 8)
Boundaries vis-à-vis the ‘outside’ world and one's own illness (n = 7)	Sheltering boundaries (n = 4)
	Oppressive boundaries (n = 4)

The participants offered differing accounts of their experiences of the inpatient ward. Some accounts could be characterized as generally positive, while others had a more negative tone. However, complexity and ambivalence appeared in each of the narratives: participants who spoke of feeling endangered by their fellow patients also spoke of finding connections or friendship on the ward; participants who spoke of the ward as a haven also said they contemplated terminating treatment. Considered together, the participants' narratives constituted a corpus of accounts that captured the varied, and sometimes starkly opposed, ways in which patients experienced life in the inpatient ward, despite the similar institutional and social conditions it presented.

### A sense of recognition and legitimacy

A valued part of being admitted into, and receiving care in, the inpatient ward was the sense that clinical authorities affirmed one's eating disordered identity. However, participants also expressed disappointment at being addressed as part of an eating disordered collective. They were therefore ambivalent about the ward's continuous definitional practices, and the groupings they created, which they simultaneously wanted and opposed.

#### The eating disordered self

As narrated by the participants, the sense of legitimacy and recognition conferred by clinical authorities encompassed both the *fact* of hospitalization, and the *experience* of being treated in an inpatient unit. To call oneself ‘anorexic’, as one participant, Vered, explained, was to lay claim to a ‘title’ – one that had to be earned. In her narrative, Vered directly compared her ‘title’ of anorexia nervosa to an academic degree (in Hebrew, the word *to'ar* is used for both); and much like an academic degree, her eating disorder had to be certified by an authoritative body. Turning to the author, she said,

Just like you, you know, worked really hard, [and] with blood, sweat, and tears got those two degrees of yours, [then] I'm not worthy of this title, I didn't spend enough on it… not enough blood, not enough tears, not enough suffering. (Vered)

Although eating disorder ‘titles’ could be conferred by any clinical authority, admission into inpatient care provided substantial recognition. For those participants who had been hospitalized, inpatient hospitalization itself emerged as a form of ‘naming’ and recognition of their eating disorder and their suffering. Narrating her initial admission into the eating disorders ward, Tali said that, after receiving her notice of ‘acceptance’,

I told my mom I didn't want to eat, so I would come [to the ward] as thin as possible… because what if they don't accept me? What if I gained a kilo? (Tali)

This quote underscores both the tenuousness of Tali's perceived status as a ‘legitimate’ anorectic, and the extent to which she sought the recognition provided by inpatient care. Along similar lines, when the author asked Emily if she defined herself as anorexic, she used the fact of inpatient treatment to quell her doubts about being a ‘good anorexic’, employing the clinicians’ judgment as arbiter of truth:

… on the one hand, I'm not thin enough/don't starve myself enough/don't-know-what enough to be anorexic.... [but] on the other hand you say, well, there's no way I'm doing this, like, on purpose… and, after all, I was hospitalized twice at [the eating disorders ward], so there's probably something there. And then you say, alright, maybe I am. (Emily)

Eating disordered identities were subject to constant negotiation. They were ‘slippery’ identities – not simply achieved and established, but dependent on continuous justification and work. Hospitalization became part of this identity work.

#### The patient as an individual

While admission into inpatient care provided authoritative recognition, a sense of legitimized identity involved the continuous acknowledgment of the patient as an individual – not merely as a bearer of an eating disorder diagnosis. For Grace, who had been hospitalized for five months continuously, the sense that clinical staff tacitly understood her was part of this legitimizing process:

They knew about me much more than I knew about myself… things that even I wasn't aware of, but that they could see from the outside.... It always gave me a good feeling – that I don't have to talk and they still know. (Grace)

Like the other participants who provided positive descriptions of care at the ward, Grace alluded to interactions with staff in which she received individualized care. In part, she said, the clinical staff's understanding of her as an individual was informed by their understanding of her as a sufferer of anorexia nervosa and obsessive-compulsive disorder. However, as other participants' accounts revealed, such diagnostically-informed understandings were also associated with experiences of being de-legitimated. Specifically, in those accounts, the sense of being treated as part of a collective rather than as an individual emerged as critical component. When Zoey explained why she deemed her hospital stay unsuccessful, she said she felt lumped together with other patients:

The specifics, they don't relate to in [the ward]…. The specifics of the individual experience that you are having as a person. They take you as like, you know, you have the symptoms here, you all have them. (Zoey)

Elsewhere in her narrative, Zoey related the sense of validation she felt at intake, when clinicians at the ward told her she was diagnosed as anorexic. However, during treatment, she felt the diagnosis overshadowed who she was as a person. Critiquing the staff's lack of attention to ‘specifics’, Zoey drew parallels between the binaries of collective (eating disordered) versus individual (self), and superficial (symptom-focused) versus deep (experience-focused) treatment.

For some participants, the sense of being treated as part of an eating disordered collective and de-legitimated as an individual became tangible in specific episodes of treatment. Danielle, who had a gastrointestinal emergency while hospitalized for her eating disorder, expressed frustration at having been mistrusted when she first complained to staff:

When I was at [the ward] and I had a [GI condition] and it was really painful, and they told me, no, it's psychological because they raised your [calorie intake].... They didn't believe me until they found me, fainted.... That's what annoys me, that they don't trust you. (Danielle)

Danielle said that, in hospital, she felt her entire experience – physical and psychological – had been subsumed under the umbrella of her eating disorder: having been told that her disorder was ‘talking’ (cf. Malson et al., 2004; Lavis, 2011 [22] [23]), her own voice had been consistently ignored.

A different form of feeling unheard was described by Emily. Following her first hospitalization at the eating disorders ward – an experience she said led to substantial gains in her everyday life, despite her trenchant eating disorder – Emily decided to return to hospital, with the goal, she said, of strengthening herself before starting university studies. During this subsequent hospitalization, however, she received treatment which she felt did not match or support her goals:

I was connected to the nasogastric tube again… and I said, this is not the reason I came here. I came so I could learn to live with [anorexia]…. And then they didn't want me to study [in university], they told me it was a mistake, that I'm not allowed to study, that I need to focus on surviving. I told them that if they think survival is my end [purpose], I prefer to die now. (Emily)

Use of nasogastric feeding was part of the treatment protocol for those underweight patients who could not consume the necessary amount of calories otherwise. But, for Emily, the clinicians' decision to resort to the nasogastric tube was a sign of misalignment between who she was as an individual and how she was perceived as a patient, a contrast between her goal of living (‘learn[ing] to live with’ her eating disorder and pursuing her studies) and the clinicians' encouragement that she focus on surviving.

### Dynamics within the inpatient community

The participants expressed ambivalence about the unique social environment constructed by the ward, where they shared their everyday lives with twenty other people with eating disorders. Although participants said they found social belonging on the ward, they also spoke of this belonging as putting them at risk for over-identification and intensified illness.

#### Seeing the self in others

The experience of encountering, interacting, and indeed living with a group of people with eating disorders was central to participants' accounts of inpatient settings. As participants described it, being hospitalized alongside fellow sufferers was a formative experience, one in which the previous solitude of living with an eating disorder was replaced by community. For some participants, the inpatient community functioned as a core element of treatment. As Alon explained,

[g]etting there, and sitting in groups, and hearing people talking about things that you're also going through – there's something very powerful in this, in this sense of ‘I'm not alone'… [we] feel like, we're all dealing here with a similar demon, and there's some sense of shared destiny. (Alon)

The power of this patient community, however, went beyond group therapy. For Alon, as for other participants, inpatient treatment provided a space for making close and lasting friendships that continued for years after his time at the ward. Such friendships were created and cultivated through a sense of camaraderie – forged not only by the shared experience of inpatient experience, but also, and more importantly, through the shared practices, perceptions, and sensations of eating disorders:

I always said that when you're sitting at the dining hall and you're spreading a bit of your cheese on the edge of the plate… a person who has an eating disorder will notice and understand it. And that is what I loved so much there, that everyone spoke my language…. Beforehand, I felt so alone, because I felt that no one understood what I was feeling, no one understood what I was saying, no one thought like I did. (Grace)

With this seemingly mundane dining hall scene, Grace conveyed a sense of connection with the patient community that extended beyond speech and toward embodied practice – a shared way of being-in-the-world. Recognizing her own experience in the gestures and words of others, Grace said she felt mutual understanding and belonging for the first time in her adolescent and adult life.

Central to the sense of connection participants found at the ward was the experience of seeing the self in others. For Alon, this experience inhered in a sense of ‘shared destiny’; for Grace, it was in shared embodied practice. While other studies on eating disorders discussed the importance of finding connections within an eating disordered community, whether in clinical settings (Rich, 2006 [7]; Smith, 2012 [6]) or online (Dias, 2003 [24]), the experience of seeing the self in others extended beyond than the context of feeling connected – it was also part of altering one's own self-concept. Emily explained that she could reconsider her own reality through interacting with other patients:

[Y]ou see the frustration in seeing an amazing, lovely girl, where there's nothing, nothing bad to say about her, and she's just ruining her life over nonsense…. And you suddenly get what other people who talk to you are going through. (Emily)

Other patients were not merely ‘mirrors’ to Emily's own reality. Rather, she was able to recognize shared practices and experiences, and then situate herself apart from them. The presence of other women with eating disorders allowed Emily to alter her own positioning, to become an observer of the ‘other’ – and, by extension, of the self-as-other – and thereby begin to think of her eating disorder as a condition to externalize.

### Dangerous others

Seeing the self in others, however, did not always promote a sense of wellbeing; for some participants, the very ‘others’ with whom they were linked posed a source of danger from within. More than half of the participants said they felt triggered by being in close proximity to very ill people, observing their appearance, and witnessing their everyday practices. For some, this triggering effect was expressed in the acquisition of new eating disordered practices. Aya, who had been hospitalized for anorexia nervosa, restricting type, described herself as ‘relatively normal’ compared to her fellow patients, and said she ‘learned’ food-related ritualistic behaviours at the ward, ‘because you see everyone making rituals and I felt very strange’. After she was discharged, she began vomiting to purge herself – a development she also related to the patient community: ‘it's something I learned… there was a reason that it seemed kind of alright to try [it] when I got out of there'.

Whereas Aya related her acquisition of eating disordered practices to striving for belonging in the patient community, other participants said the community triggered disorder-supportive desires. Natalie, who was hospitalized for anorexia nervosa, said she felt triggered by the very sight of emaciated patients:

When you reach a condition that's relatively healthy and fine and you're halfway there… suddenly a girl who weighs 20 kilos shows up… I don't want to see it…. It's not that it's the sick side [of me], it's like – it's the side I never had. So why do I need to get acquainted with it? (Natalie)

Earlier in her narrative, Natalie said that, while she used to think her thoughts and practices were unique, upon entering treatment, she discovered that others had lived through the exact same experiences. She also said she developed close friendships with some of her fellow patients. Yet, the same connections which brought together the patient community also implicated kinship between Natalie and the critically ill – a kinship which Natalie felt endangered her own drive to recovery by suggesting possibilities, and indeed awakening desires for extremes of eating disorder which she had not yet experienced.

Like Natalie, other participants said they felt deeply unsettled when confronted with the varieties (particularly the extremes) of the eating disordered experience. As the ward provided patients with a reference group, it also, by extension, provided them with a new range of possibilities for eating disordered practice. For example, Danielle said she felt endangered in the group when other women spoke of suicide attempts and desire for eating disorders: ‘I felt I was being dragged back more than going forward’. Vered said she felt her own claim to illness was de-legitimized when one patient told her of a former patient who worked as a fitness instructor while starving herself, but continued to feel strong: ‘I suddenly began to feel jealous, like, it's not enough that I reached this weight and did all that, now I have another goal, I also have to mortify myself and feel strong despite everything'. And Yif'at, who said eating disorder concepts were ‘dripping into [her] brain unconsciously', said that being exposed to a variety of motivations for eating disorders at the ward allowed her to change her own motivations – from desire for disappearance to desire for slimness – and thereby sustain her practice by adopting a concept of eating disorder more commensurate with her current lifestyle. The participants, notably, did not say they actively sought out ‘tips and tricks' from other patients (cf. Rich, 2006 [7]); rather, it was in the intimacy of living with eating disordered others that they began to embody their practices and logics.

### Boundaries vis-a-vis the ‘outside’ world and one's own illness

The participants described the ward's setting of highly regimented schedules and practices as creating a special space, with boundaries that separated the patients from external forces. However, participants expressed ambivalence about the meaning of these boundaries, which they alternately identified as sheltering and as oppressive.

#### Sheltering boundaries

A typical stay in inpatient care entailed weeks or months of living in the ward. Patients who showed progress were allowed to go home for weekends, or spend a weekday afternoon away; for the most part, however, inpatient stays involved continuous living in the ward's enclosed space, with the occasional supervised excursion to the ward's front yard, or a guided walk at night to a nearby landmark. Life on the ward was marked by the strict schedules and regimens of diet and activity prescribed by the senior clinicians, under the observation of nursing staff. For some, this regimented living was a source of comfort:

There is something really good about the fact that there's an organized diet, that there's some sort of certainty…. Things are very clear. And it spares you this engagement, a certain part of the engagement that used to exist at an obsessive level. (Alon)

As Alon described it, the ward's dietary regime provided a ‘clear’ anchor, a means of letting go of obsessive engagements with food. His eating disordered practice, then, was displaced away from his daily routine and into a designated space with its own rules. Alon added, however, that months after completing his hospital stay, when facing the choice of whether or not to be hospitalized a second time, he decisively chose outpatient treatment in the community.

For other participants, it was not only the ward's regime that provided comfort, but also the ward's very separation from the outside world – spatially, temporally, and relationally. Tali, who continued to reminisce about her time on the ward years after she had been hospitalized, said:

I didn't want to leave, I didn't want to leave, no one wanted to leave… as difficult as it was, there were many difficult things, but – but it was sort of a greenhouse. (Tali)

In Israel, the word ‘greenhouse’ is a commonly used descriptor for places or situations that care for, and perhaps overprotect, their (often young) charges. In using ‘greenhouse’ to describe the ward, Tali alluded to its status as a bubble, bounded and sheltering from the outside world, yet not sustainable for the long term. Notably, although Tali said she did not want to leave, elsewhere in her narrative, she also related an anecdote of a threat she had made, early in her hospitalization, to leave the ward – after which, Tali said, she chose to remain in hospital. Along similar lines, when Grace described the ward, she chose the word ‘lab’:

It was a little lab like that, that you could be inside…. A lab in the sense that it was very sterile, it was – very very exact and measured conditions, and – you knew that you, it's not like the real world, so it eased [our burden]. (Grace)

Through its precision, regimented living, and inward-looking ethos, the ward, for Grace, provided a time and space apart. At the same time, Grace said she kept her suitcase packed for the entire duration of her inpatient stay: ‘I was always with one foot out’. Like Grace and Tali, Meital, who had been hospitalized twice, said she did not want to leave the ward following her first inpatient stay. Meital, however, described the ward neither as a ‘greenhouse’ nor as a ‘lab’, but as a platform for maturing and individuating:

The first hospitalization (laughs) – it funny to say but I enjoyed it. Like, suddenly I had friends, and it was really pleasant, and it was also, somehow, [a way of] getting out of home, something that I wanted. I wanted my privacy and my independence and I had it there. (Meital)

The ward, as Meital described it, provided a space and time for accelerated growth; within its walls, she found not only a group of friends, but also boundaries from her family home.

#### Oppressive boundaries

While some participants cited the ward's boundaries as providing shelter and comfort, those same boundaries also emerged as oppressive in other accounts. As discussed above, Meital described her first hospitalization as a positive experience that provided her with space for growth. When she returned to the ward a second time, however, her sense of the ward's boundaries had inverted:

The second hospitalization, in comparison, was very traumatic. I felt really bad there. I couldn't find myself… Being in a closed ward with very tough discipline, very clear rules, where they decide for you when you'll eat, when you'll have time for breaks, like – it didn't suit me anymore. I needed my freedom, to decide on my own structure. (Meital)

Although the ward itself had remained the same, what changed, according to Meital, was her emerging desire for freedom. The independence she had craved – and which she felt the first hospitalization provided her – now led her to experience the ward as oppressive. Indeed, some participants' perceptions of ward life were linked to their sense of maturity. For example, Zoey, who was in her 30 s when she entered the ward, explained that, unlike other patients, she had ‘achieved things’ and ‘somehow had combined doing my adult life and being sick’; when she described her experience of ward regimes, she said: ‘[i]t was a horrible programme for me – forcing me to do anything is not good for me’. It should be noted, however, that Zoey chose to complete her treatment programme in full.

Like Zoey, Meital, and other participants, Vered described the rules of the ward, and the ward's very confines, as disempowering. Vered, who had an infant daughter, said she felt dispirited and exhausted by the treatment regime:

Even though I feel that I progress a lot and such, I'm really sick of this.… It's very very difficult for me, this distance from [my daughter], from my home…. Being here, eating this diet, I have no strength anymore, I'm tired already.(Vered)

Unlike some of the younger patients, Vered felt strongly linked and committed to the outside world through her daughter, such that the ward's regime, and her abiding by it, became oppressive, alien, and even absurd – ‘eating this diet’ juxtaposed to separation from her infant child.

### Discussion

The participants expressed ambivalence about the identities, social environment, and regimented living constructed by the ward. In their narrated experiences of inpatient care, participants described clinical recognition of one's eating disorder as both affirming and reductive, fellow patients as both supportive and dangerous, and the boundaries of the ward as both protective and oppressive. These themes were consistent with findings from previous studies on the experiences of inpatients in eating disorders wards. This is particularly notable given that this study is the first to examine inpatient experiences in Israel, with previous studies having been situated in the United Kingdom. Gooldin (2002 [13]) argues that Israeli patients' experiences of eating disorders differ from those of patients in Europe and North America, and reflect identity concerns specific to Israel, namely military service and pro-natalism. Moreover, as this study's author argues elsewhere (Eli, 2014 [25]), compulsory military service in young adulthood – an experience that distinguishes Israeli women from their Euro-American peers – can impact the development of Israeli women's eating disorders. This study, however, demonstrates that Israeli patients' experiences are similar to those of British patients, suggesting that inpatient environments share cross-cultural commonalities in their social and institutional dynamics.

While this study supports findings from other published studies, the analysis of ambivalence as embedded in the social and institutional setting of the inpatient ward highlights the patients' central concern with difference and belonging – a concern that has not been addressed previously. Colton and Pistrang (2004 [3]), Offord et al. (2006 [4]), and Smith (2012 [6]) all identify individualized care as important, and note the conflict between feeling supported and feeling triggered by other patients; however, they interpret their findings through the lens of patient-specific circumstances, motivations, and processes. As this study demonstrates, interpreting the experience of individual patients as contextualized in the treatment milieu they encounter can help explain why ambivalence is pervasive among inpatients, individual factors notwithstanding. Notably, in this study, participants simultaneously defined themselves as part of, but also distinct from, the eating disordered patients who formed their reference group at the ward, negotiating a protectively ambivalent positioning. Through this ambivalent positioning, participants affirmed their eating disordered identities and connected with others on the ward, while also asserting their non-disordered individuality and distancing themselves from the potential dangers posed by ‘excessive’ belonging.

This ambivalent positioning could usefully be understood through the anthropological concept of *liminality* – being ‘betwixt and between’ (Turner, 1967 [1964] [26]). Originally formulated to define the position of the adolescent initiand in a rite-of-passage, the concept of liminality was used to describe a period of transition between childhood and adulthood, navigated by a person of ambivalent social status (Van Gennep, 1960 [1909] [27]). However, liminality has since been applied in wide-ranging anthropological analyses of the individual, community, time, and space, such that it now describes not only persons-in-transition, but also indefinitely marginal and ambiguous states.

Some of the experiences described by the study's participants evoked the liminality of the ward itself. In the participants' accounts, the ward emerged as having ambiguous status – a ‘greenhouse’ or ‘lab’; a space with its own rules, contained by, but also separate from, the world outside. Indeed, the concept of liminality has been employed in several ethnographic and other qualitative analyses of hospital life. The hospital itself, being a space of constant transitions, both geographic (from one ward to another) and existential (birth, critical illness, survival, death), has been analyzed as liminal – set apart from the ‘outside’ world, located at the margins of defined being. Several analyses have explored how the hospital, as a liminal space, creates distinct land- and sound-scapes of clinical care – alternative sensory worlds that permeate one's own experience and self-definition as a patient (Rice, 2003; Van der Geest and Finkler, 2004; Long et al., 2008 [28] – [30]). Recently, the concept of liminality has also been applied to analyses of in-hospital rites-of-passage, such as the transitioning of patients from children's wards to adult wards (Tierney et al., 2013 [31]).

Another aspect of liminal experience that emerged in the participants' accounts concerned the social marginality of eating disorder, and specifically the connections they found with fellow patients who could tacitly understand their experiential, sensory, and emotional worlds. In ethnographic work on chronic illness, states of chronicity and remission have been analyzed as liminal being: constituting a ‘biographical disruption' in a person's life (Bury, 1982 [32]), challenging self-concepts, and placing the ill person, her body, and her embodied experience on the margins of ‘healthy’ society (Little et al., 1998; Charmaz, 2002; Jackson, 2005; Frank, 2010 [33] – [36]).

While the dual liminality of eating disorder and of the ward space – both positioned as marginal to the ‘outside’ world – was an important feature of the narratives, this study suggests that the form of liminality most fundamental to the participants' accounts concerned the ambivalent positioning they negotiated vis-a-vis the ward and its inhabitants. Living in an enclosed space with a group of twenty eating disordered peers, apart from non-eating-disordered people (excepting the ward's staff), the participants were confronted daily with a reference group defined by clinical authorities. This group, as their accounts demonstrated, was a source of ambivalent engagement, at once compelling and dangerous: promising recognition but also threatening one's individuality, potentiating friendship and de-marginalization but also the possibility of further illness and disconnection from the ‘outside’.

The participants' negotiation of liminal identities – both a *part of*, and *apart from*, the patient community – was expressed in their experiences of treatment. While being recognized as eating disordered was a validating aspect of treatment, being treated as part of an eating disordered collective was not. Likewise, while participants spoke positively of finding connections on the ward, seeing the self in eating disordered others could pose dangerous possibilities for illness. And while the ward's structure and rules could offer positive detachment from the exigencies of daily life, those same structures and rules could be oppressive when they trampled on an individual's sense of independence and connectedness with the outside world.

Conflicting attitudes toward treatment, then, reflected more than patients' capacities for or ambivalence toward recovery (Williams and Reid, 2010 [8]), or their struggling between disorder-maintaining ‘voices’ and recovery-supportive ‘voices’ (Hope et al., 2011 [37]): they were embedded in the social and institutional setting of the inpatient ward, and linked to the forging of patient identities. For this study's participants, ambivalent attitudes emerged as part of life in the ward, and as a constitutive feature of negotiating the dynamics of belonging to the patient community while distinguishing oneself from it. Future work should explore the ambivalence that patients endorse as a potentially protective, rather than disruptive, strategy for navigating inpatient treatment. From a clinical practice perspective, the results suggest that directly acknowledging and normalizing feelings of ambivalence toward inpatient treatment in individual therapy or group sessions could improve provider-patient communication, and might contribute to patients' retention and completion of inpatient treatment.

The study had several limitations. With the great majority of participants, interviews took place years after their treatment at the inpatient ward had ended, such that their narrated experiences of hospitalization were retrospective. Moreover, as inpatient hospitalization was not the central focus of the study, and as the interview style allowed for variations according to each participant's engagement, the length and depth of the participants' accounts of inpatient experience varied; two of the 13 participants provided only cursory accounts of their inpatient experiences, and the narratives of the other eleven participants had to be privileged in the analysis. In addition, while all participants in the subsample of 13 had been hospitalized in the same adult eating disorders ward, there were considerable variations in their length of stay, number of past hospitalizations, and other experiences of inpatient and outpatient care. And, while the ward provided inpatient treatment for the entire spectrum of eating disorders, including bulimia nervosa and binge eating disorder, all but one of the 13 had been hospitalized for anorexia nervosa or its subclinical variants. It should also be noted that the author was acquainted with one of the thirteen participants before beginning this research project – a common occurrence in anthropology. Although the author did not know the details of this participant's eating disorder experience prior to the study, this participant's interview process may have been influenced by some foreknowledge. Finally, it is important to highlight that the data collection and analysis were conducted by a lone researcher; while this is an accepted and widespread practice in anthropology, it does preclude validity checking through intercoder reliability.

### Conclusion

This study examined the narrated experiences of 13 people who had been hospitalized in a dedicated eating disorders ward for adults in central Israel. Focusing on inpatient experiences of ambivalence as embedded in the social and institutional setting of the ward, the analysis highlighted the participants' central concern with difference and belonging. The participants simultaneously defined themselves as part of, but also distinct from, the eating disordered patients who formed their reference group at the ward. Through this ambivalent positioning, participants could affirm their eating disordered identities and connect with others on the ward, while also asserting their non-disordered individuality and distancing themselves from the potential dangers posed by ‘excessive’ belonging. The findings suggest that future research should explore inpatients' ambivalence as potentially protective, rather than disruptive, and that directly acknowledging and normalizing inpatients' feelings of ambivalence might benefit patient-provider communication and treatment programme completion.
